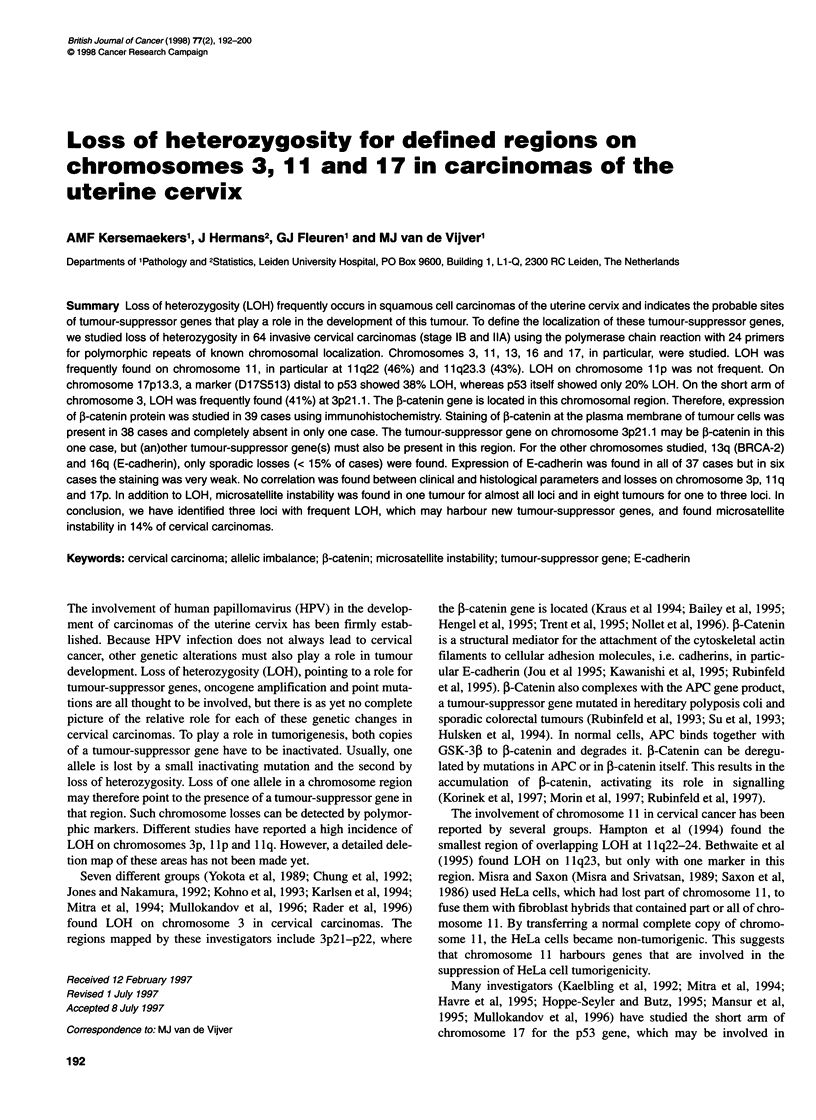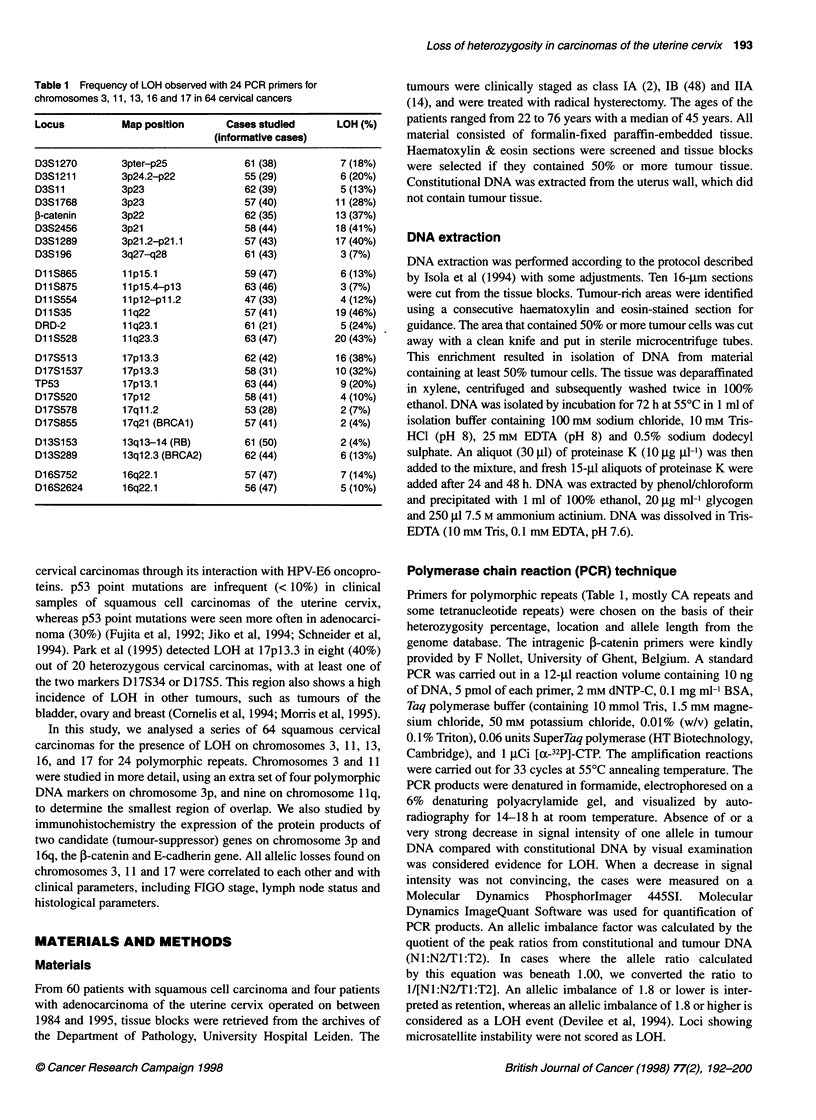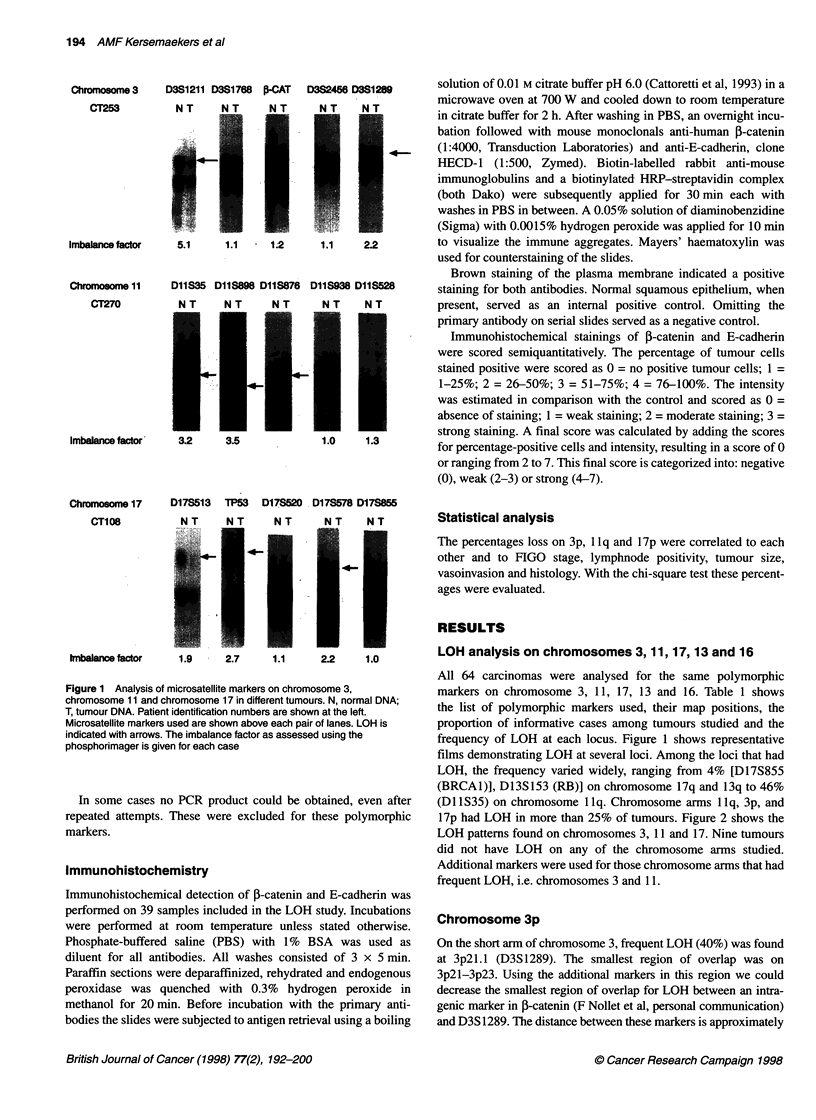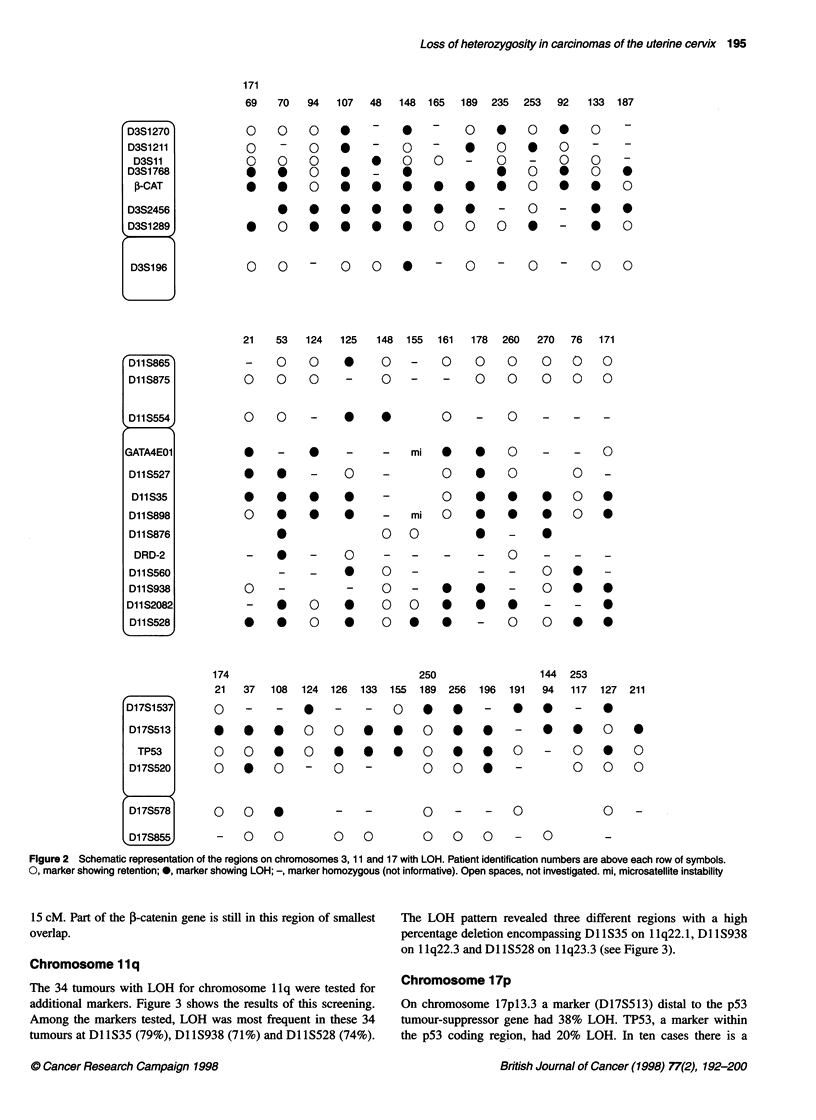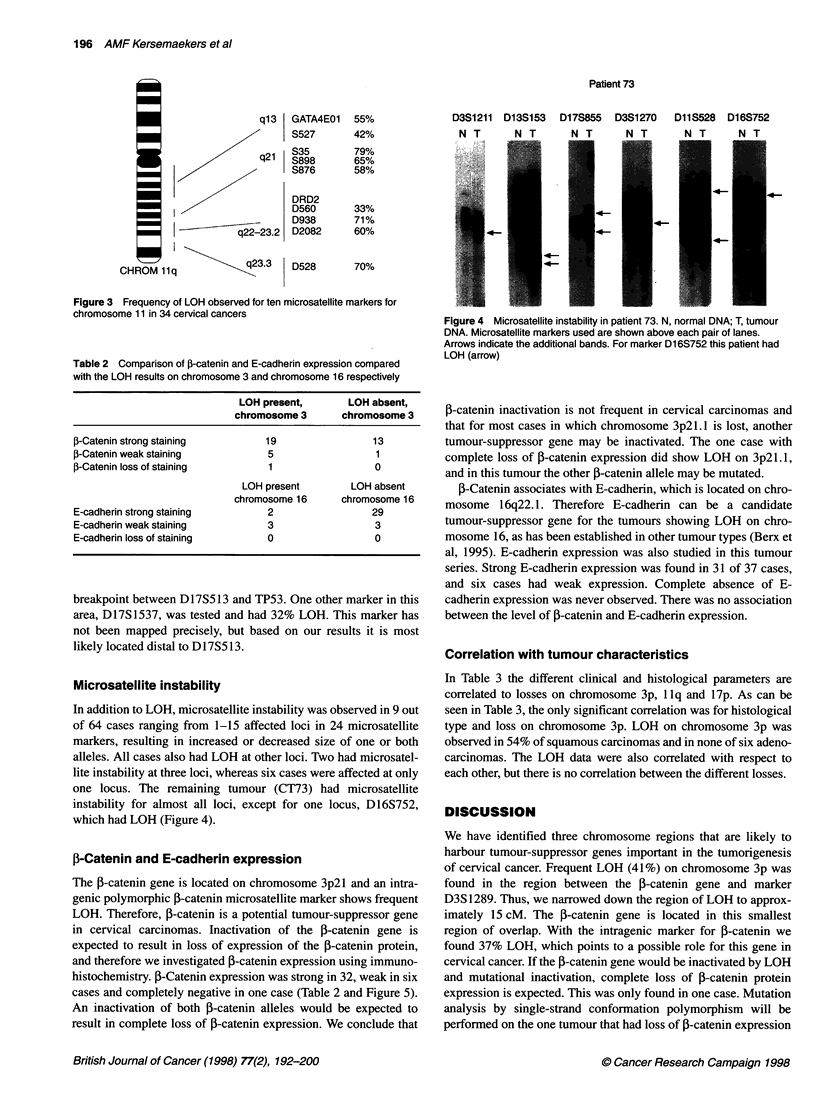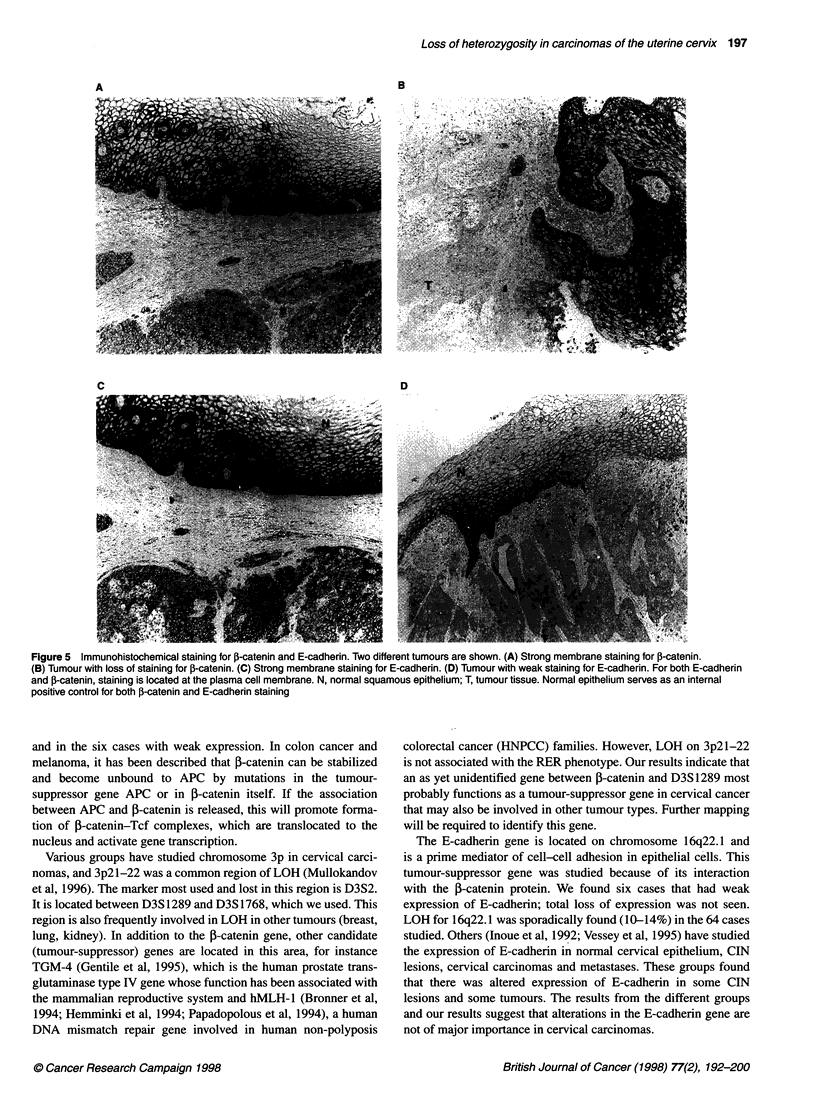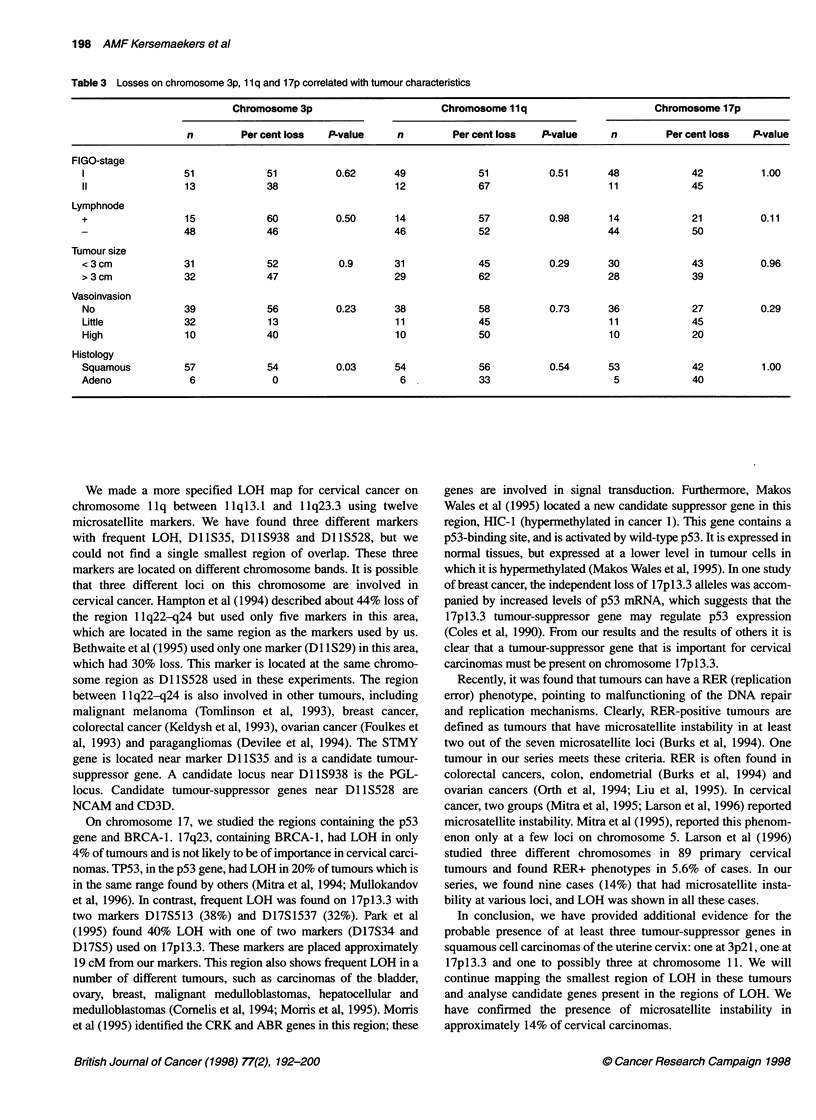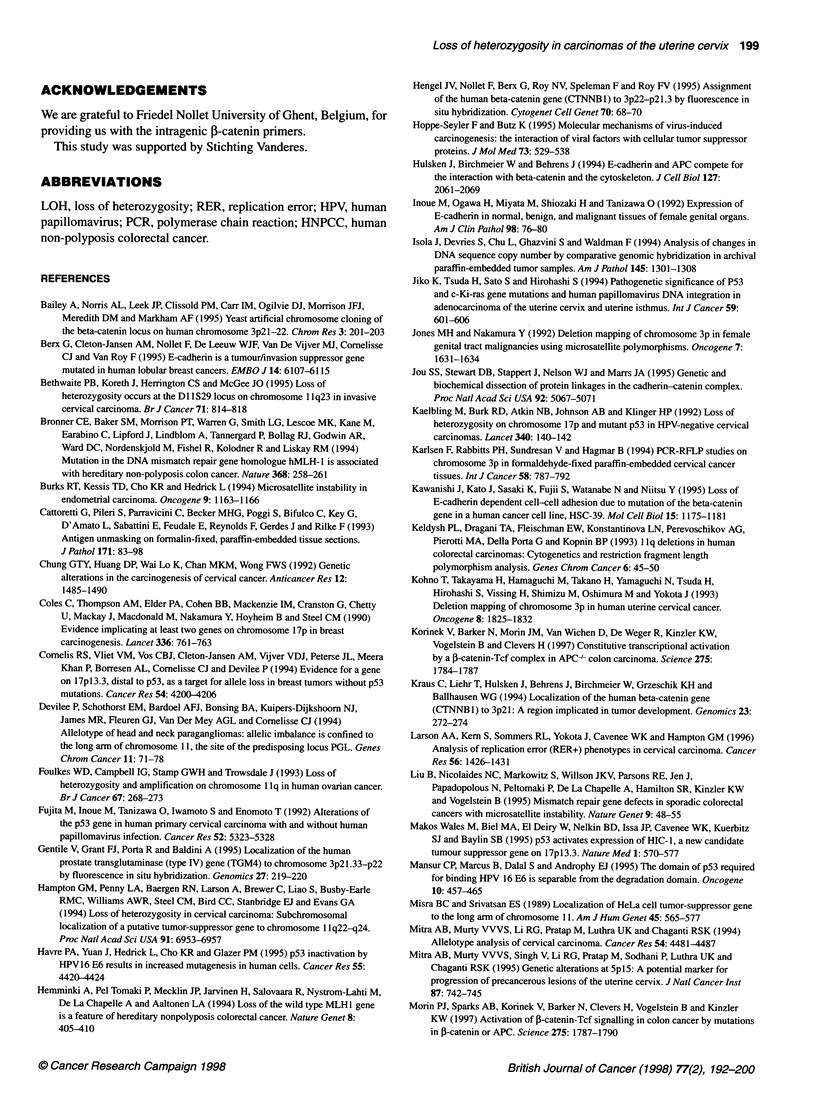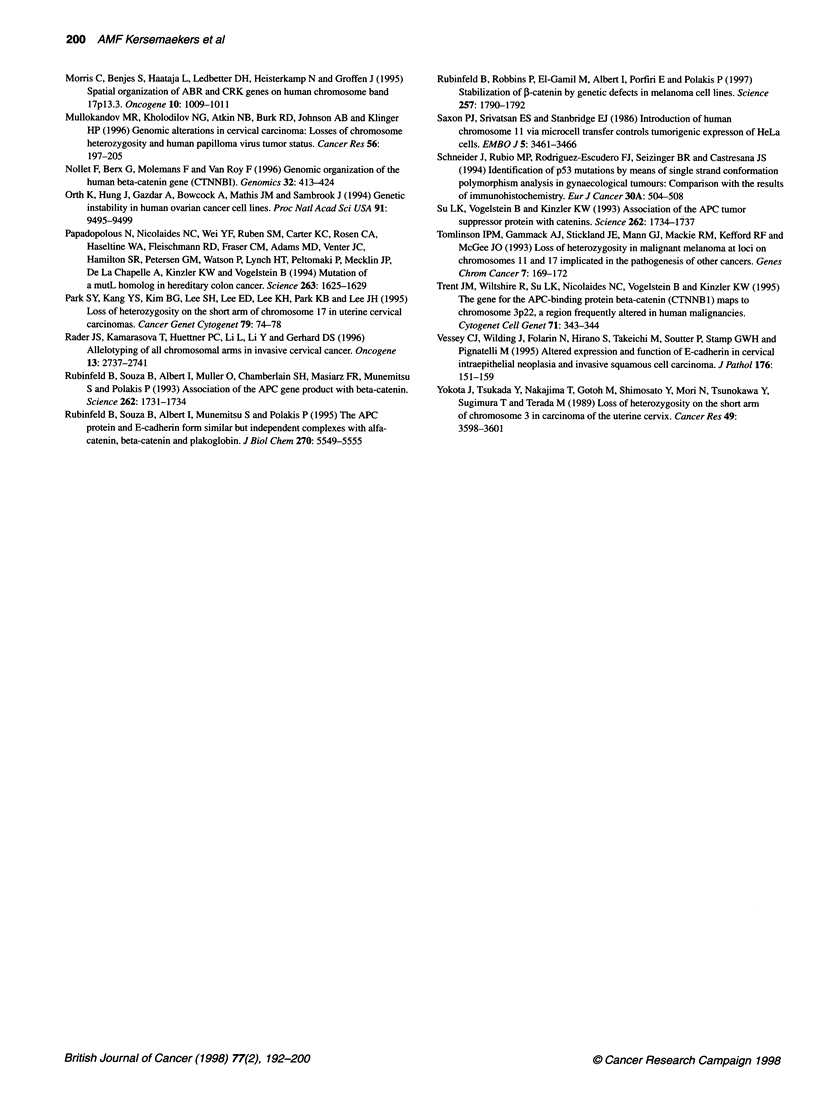# Loss of heterozygosity for defined regions on chromosomes 3, 11 and 17 in carcinomas of the uterine cervix.

**DOI:** 10.1038/bjc.1998.33

**Published:** 1998

**Authors:** A. M. Kersemaekers, J. Hermans, G. J. Fleuren, M. J. van de Vijver

**Affiliations:** Department of Pathology, Leiden University Hospital, The Netherlands.

## Abstract

**Images:**


British Journal of Cancer (1998) 77(2), 192–200
© 1998 Cancer Research Campaign

### Loss of heterozygosity for defined regions on chromosomes 3, 11 and 17 in carcinomas of the uterine cervix

**AMF Kersemaekers[1], J Hermans[2], GJ Fleuren[1] and MJ van de Vijver[1]**

Departments of [1]Pathology and [2]Statistics, Leiden University Hospital, PO Box 9600, Building 1, L1-Q, 2300 RC Leiden, The Netherlands

**Summary** Loss of heterozygosity (LOH) frequently occurs in squamous cell carcinomas of the uterine cervix and indicates the probable sites of tumour-suppressor genes that play a role in the development of this tumour. To define the localization of these tumour-suppressor genes, we studied loss of heterozygosity in 64 invasive cervical carcinomas (stage IB and IIA) using the polymerase chain reaction with 24 primers for polymorphic repeats of known chromosomal localization. Chromosomes 3, 11, 13, 16 and 17, in particular, were studied. LOH was frequently found on chromosome 11, in particular at 11q22 (46%) and 11q23.3 (43%). LOH on chromosome 11p was not frequent. On chromosome 17p13.3, a marker (D17S513) distal to p53 showed 38% LOH, whereas p53 itself showed only 20% LOH. On the short arm of chromosome 3, LOH was frequently found (41%) at 3p21.1. The β-catenin gene is located in this chromosomal region. Therefore, expression of β-catenin protein was studied in 39 cases using immunohistochemistry. Staining of β-catenin at the plasma membrane of tumour cells was present in 38 cases and completely absent in only one case. The tumour-suppressor gene on chromosome 3p21.1 may be β-catenin in this one case, but (an)other tumour-suppressor gene(s) must also be present in this region. For the other chromosomes studied, 13q (BRCA-2) and 16q (E-cadherin), only sporadic losses (< 15% of cases) were found. Expression of E-cadherin was found in all of 37 cases but in six cases the staining was very weak. No correlation was found between clinical and histological parameters and losses on chromosome 3p, 11q and 17p. In addition to LOH, microsatellite instability was found in one tumour for almost all loci and in eight tumours for one to three loci. In conclusion, we have identified three loci with frequent LOH, which may harbour new tumour-suppressor genes, and found microsatellite instability in 14% of cervical carcinomas.

**Keywords:** cervical carcinoma; allelic imbalance; β-catenin; microsatellite instability; tumour-suppressor gene; E-cadherin

The involvement of human papillomavirus (HPV) in the development of carcinomas of the uterine cervix has been firmly established. Because HPV infection does not always lead to cervical cancer, other genetic alterations must also play a role in tumour development. Loss of heterozygosity (LOH), pointing to a role for tumour-suppressor genes, oncogene amplification and point mutations are all thought to be involved, but there is as yet no complete picture of the relative role for each of these genetic changes in cervical carcinomas. To play a role in tumorigenesis, both copies of a tumour-suppressor gene have to be inactivated. Usually, one allele is lost by a small inactivating mutation and the second by loss of heterozygosity. Loss of one allele in a chromosome region may therefore point to the presence of a tumour-suppressor gene in that region. Such chromosome losses can be detected by polymorphic markers. Different studies have reported a high incidence of LOH on chromosomes 3p, 11p and 11q. However, a detailed deletion map of these areas has not been made yet.

Seven different groups (Yokota et al, 1989; Chung et al, 1992; Jones and Nakamura, 1992; Kohno et al, 1993; Karlsen et al, 1994; Mitra et al, 1994; Mullokandov et al, 1996; Rader et al, 1996) found LOH on chromosome 3 in cervical carcinomas. The regions mapped by these investigators include 3p21–p22, where the β-catenin gene is located (Kraus et al 1994; Bailey et al, 1995; Hengel et al, 1995; Trent et al, 1995; Nollet et al, 1996). β-Catenin is a structural mediator for the attachment of the cytoskeletal actin filaments to cellular adhesion molecules, i.e. cadherins, in particular E-cadherin (Jou et al 1995; Kawanishi et al, 1995; Rubinfeld et al, 1995). β-Catenin also complexes with the APC gene product, a tumour-suppressor gene mutated in hereditary polyposis coli and sporadic colorectal tumours (Rubinfeld et al, 1993; Su et al, 1993; Hulsken et al, 1994). In normal cells, APC binds together with GSK-3β to β-catenin and degrades it. β-Catenin can be deregulated by mutations in APC or in β-catenin itself. This results in the accumulation of β-catenin, activating its role in signalling (Korinek et al, 1997; Morin et al, 1997; Rubinfeld et al, 1997).

The involvement of chromosome 11 in cervical cancer has been reported by several groups. Hampton et al (1994) found the smallest region of overlapping LOH at 11q22–24. Bethwaite et al (1995) found LOH on 11q23, but only with one marker in this region. Misra and Saxon (Misra and Srivatsan, 1989; Saxon et al, 1986) used HeLa cells, which had lost part of chromosome 11, to fuse them with fibroblast hybrids that contained part or all of chromosome 11. By transferring a normal complete copy of chromosome 11, the HeLa cells became non-tumorigenic. This suggests that chromosome 11 harbours genes that are involved in the suppression of HeLa cell tumorigenicity.

Many investigators (Kaelbling et al, 1992; Mitra et al, 1994; Havre et al, 1995; Hoppe-Seyler and Butz, 1995; Mansur et al, 1995; Mullokandov et al, 1996) have studied the short arm of chromosome 17 for the p53 gene, which may be involved in

*Received 12 February 1997*
*Revised 1 July 1997*
*Accepted 8 July 1997*

*Correspondence to:* MJ van de Vijver

**Table 1** Frequency of LOH observed with 24 PCR primers for chromosomes 3, 11, 13, 16 and 17 in 64 cervical cancers

| Locus | Map position | Cases studied (informative cases) | LOH (%) |
|---|---|---|---|
| D3S1270 | 3pter–p25 | 61 (38) | 7 (18%) |
| D3S1211 | 3p24.2–p22 | 55 (29) | 6 (20%) |
| D3S11 | 3p23 | 62 (39) | 5 (13%) |
| D3S1768 | 3p23 | 57 (40) | 11 (28%) |
| β-catenin | 3p22 | 62 (35) | 13 (37%) |
| D3S2456 | 3p21 | 58 (44) | 18 (41%) |
| D3S1289 | 3p21.2–p21.1 | 57 (43) | 17 (40%) |
| D3S196 | 3q27–q28 | 61 (43) | 3 (7%) |
| D11S865 | 11p15.1 | 59 (47) | 6 (13%) |
| D11S875 | 11p15.4–p13 | 63 (46) | 3 (7%) |
| D11S554 | 11p12–p11.2 | 47 (33) | 4 (12%) |
| D11S35 | 11q22 | 57 (41) | 19 (46%) |
| DRD-2 | 11q23.1 | 61 (21) | 5 (24%) |
| D11S528 | 11q23.3 | 63 (47) | 20 (43%) |
| D17S513 | 17p13.3 | 62 (42) | 16 (38%) |
| D17S1537 | 17p13.3 | 58 (31) | 10 (32%) |
| TP53 | 17p13.1 | 63 (44) | 9 (20%) |
| D17S520 | 17p12 | 58 (41) | 4 (10%) |
| D17S578 | 17q11.2 | 53 (28) | 2 (7%) |
| D17S855 | 17q21 (BRCA1) | 57 (41) | 2 (4%) |
| D13S153 | 13q13–14 (RB) | 61 (50) | 2 (4%) |
| D13S289 | 13q12.3 (BRCA2) | 62 (44) | 6 (13%) |
| D16S752 | 16q22.1 | 57 (47) | 7 (14%) |
| D16S2624 | 16q22.1 | 56 (47) | 5 (10%) |

cervical carcinomas through its interaction with HPV-E6 oncoproteins. p53 point mutations are infrequent (< 10%) in clinical samples of squamous cell carcinomas of the uterine cervix, whereas p53 point mutations were seen more often in adenocarcinoma (30%) (Fujita et al, 1992; Jiko et al, 1994; Schneider et al, 1994). Park et al (1995) detected LOH at 17p13.3 in eight (40%) out of 20 heterozygous cervical carcinomas, with at least one of the two markers D17S34 or D17S5. This region also shows a high incidence of LOH in other tumours, such as tumours of the bladder, ovary and breast (Cornelis et al, 1994; Morris et al, 1995).

In this study, we analysed a series of 64 squamous cervical carcinomas for the presence of LOH on chromosomes 3, 11, 13, 16, and 17 for 24 polymorphic repeats. Chromosomes 3 and 11 were studied in more detail, using an extra set of four polymorphic DNA markers on chromosome 3p, and nine on chromosome 11q, to determine the smallest region of overlap. We also studied by immunohistochemistry the expression of the protein products of two candidate (tumour-suppressor) genes on chromosome 3p and 16q, the β-catenin and E-cadherin gene. All allelic losses found on chromosomes 3, 11 and 17 were correlated to each other and with clinical parameters, including FIGO stage, lymph node status and histological parameters.

### MATERIALS AND METHODS

### Materials

From 60 patients with squamous cell carcinoma and four patients with adenocarcinoma of the uterine cervix operated on between 1984 and 1995, tissue blocks were retrieved from the archives of the Department of Pathology, University Hospital Leiden. The tumours were clinically staged as class IA (2), IB (48) and IIA (14), and were treated with radical hysterectomy. The ages of the patients ranged from 22 to 76 years with a median of 45 years. All material consisted of formalin-fixed paraffin-embedded tissue. Haematoxylin & eosin sections were screened and tissue blocks were selected if they contained 50% or more tumour tissue. Constitutional DNA was extracted from the uterus wall, which did not contain tumour tissue.

### DNA extraction

DNA extraction was performed according to the protocol described by Isola et al (1994) with some adjustments. Ten 16-μm sections were cut from the tissue blocks. Tumour-rich areas were identified using a consecutive haematoxylin and eosin-stained section for guidance. The area that contained 50% or more tumour cells was cut away with a clean knife and put in sterile microcentrifuge tubes. This enrichment resulted in isolation of DNA from material containing at least 50% tumour cells. The tissue was deparaffinated in xylene, centrifuged and subsequently washed twice in 100% ethanol. DNA was isolated by incubation for 72 h at 55°C in 1 ml of isolation buffer containing 100 mM sodium chloride, 10 mM Tris-HCl (pH 8), 25 mM EDTA (pH 8) and 0.5% sodium dodecyl sulphate. An aliquot (30 μl) of proteinase K (10 μg $\mu l^{-1}$) was then added to the mixture, and fresh 15-μl aliquots of proteinase K were added after 24 and 48 h. DNA was extracted by phenol/chloroform and precipitated with 1 ml of 100% ethanol, 20 μg $ml^{-1}$ glycogen and 250 μl 7.5 M ammonium actinium. DNA was dissolved in Tris-EDTA (10 mM Tris, 0.1 mM EDTA, pH 7.6).

### Polymerase chain reaction (PCR) technique

Primers for polymorphic repeats (Table 1, mostly CA repeats and some tetranucleotide repeats) were chosen on the basis of their heterozygosity percentage, location and allele length from the genome database. The intragenic β-catenin primers were kindly provided by F Nollet, University of Ghent, Belgium. A standard PCR was carried out in a 12-μl reaction volume containing 10 ng of DNA, 5 pmol of each primer, 2 mM dNTP-C, 0.1 mg $ml^{-1}$ BSA, *Taq* polymerase buffer (containing 10 mmol Tris, 1.5 mM magnesium chloride, 50 mM potassium chloride, 0.01% (w/v) gelatin, 0.1% Triton), 0.06 units Super*Taq* polymerase (HT Biotechnology, Cambridge), and 1 μCi [α-$^{32}$P]-CTP. The amplification reactions were carried out for 33 cycles at 55°C annealing temperature. The PCR products were denatured in formamide, electrophoresed on a 6% denaturing polyacrylamide gel, and visualized by autoradiography for 14–18 h at room temperature. Absence of or a very strong decrease in signal intensity of one allele in tumour DNA compared with constitutional DNA by visual examination was considered evidence for LOH. When a decrease in signal intensity was not convincing, the cases were measured on a Molecular Dynamics PhosphorImager 445SI. Molecular Dynamics ImageQuant Software was used for quantification of PCR products. An allelic imbalance factor was calculated by the quotient of the peak ratios from constitutional and tumour DNA (N1:N2/T1:T2). In cases where the allele ratio calculated by this equation was beneath 1.00, we converted the ratio to 1/[N1:N2/T1:T2]. An allelic imbalance of 1.8 or lower is interpreted as retention, whereas an allelic imbalance of 1.8 or higher is considered as a LOH event (Devilee et al, 1994). Loci showing microsatellite instability were not scored as LOH.

© Cancer Research Campaign 1998

**Figure 1** Analysis of microsatellite markers on chromosome 3, chromosome 11 and chromosome 17 in different tumours. N, normal DNA; T, tumour DNA. Patient identification numbers are shown at the left. Microsatellite markers used are shown above each pair of lanes. LOH is indicated with arrows. The imbalance factor as assessed using the phosphorimager is given for each case

In some cases no PCR product could be obtained, even after repeated attempts. These were excluded for these polymorphic markers.

### Immunohistochemistry

Immunohistochemical detection of β-catenin and E-cadherin was performed on 39 samples included in the LOH study. Incubations were performed at room temperature unless stated otherwise. Phosphate-buffered saline (PBS) with 1% BSA was used as diluent for all antibodies. All washes consisted of 3 × 5 min. Paraffin sections were deparaffinized, rehydrated and endogenous peroxidase was quenched with 0.3% hydrogen peroxide in methanol for 20 min. Before incubation with the primary antibodies the slides were subjected to antigen retrieval using a boiling solution of 0.01 M citrate buffer pH 6.0 (Cattoretti et al, 1993) in a microwave oven at 700 W and cooled down to room temperature in citrate buffer for 2 h. After washing in PBS, an overnight incubation followed with mouse monoclonals anti-human β-catenin (1:4000, Transduction Laboratories) and anti-E-cadherin, clone HECD-1 (1:500, Zymed). Biotin-labelled rabbit anti-mouse immunoglobulins and a biotinylated HRP–streptavidin complex (both Dako) were subsequently applied for 30 min each with washes in PBS in between. A 0.05% solution of diaminobenzidine (Sigma) with 0.0015% hydrogen peroxide was applied for 10 min to visualize the immune aggregates. Mayers' haematoxylin was used for counterstaining of the slides.

Brown staining of the plasma membrane indicated a positive staining for both antibodies. Normal squamous epithelium, when present, served as an internal positive control. Omitting the primary antibody on serial slides served as a negative control.

Immunohistochemical stainings of β-catenin and E-cadherin were scored semiquantitatively. The percentage of tumour cells stained positive were scored as 0 = no positive tumour cells; 1 = 1–25%; 2 = 26–50%; 3 = 51–75%; 4 = 76–100%. The intensity was estimated in comparison with the control and scored as 0 = absence of staining; 1 = weak staining; 2 = moderate staining; 3 = strong staining. A final score was calculated by adding the scores for percentage-positive cells and intensity, resulting in a score of 0 or ranging from 2 to 7. This final score is categorized into: negative (0), weak (2–3) or strong (4–7).

### Statistical analysis

The percentages loss on 3p, 11q and 17p were correlated to each other and to FIGO stage, lymphnode positivity, tumour size, vasoinvasion and histology. With the chi-square test these percentages were evaluated.

### RESULTS

### LOH analysis on chromosomes 3, 11, 17, 13 and 16

All 64 carcinomas were analysed for the same polymorphic markers on chromosome 3, 11, 17, 13 and 16. Table 1 shows the list of polymorphic markers used, their map positions, the proportion of informative cases among tumours studied and the frequency of LOH at each locus. Figure 1 shows representative films demonstrating LOH at several loci. Among the loci that had LOH, the frequency varied widely, ranging from 4% [D17S855 (BRCA1)], D13S153 (RB)] on chromosome 17q and 13q to 46% (D11S35) on chromosome 11q. Chromosome arms 11q, 3p, and 17p had LOH in more than 25% of tumours. Figure 2 shows the LOH patterns found on chromosomes 3, 11 and 17. Nine tumours did not have LOH on any of the chromosome arms studied. Additional markers were used for those chromosome arms that had frequent LOH, i.e. chromosomes 3 and 11.

### Chromosome 3p

On the short arm of chromosome 3, frequent LOH (40%) was found at 3p21.1 (D3S1289). The smallest region of overlap was on 3p21–3p23. Using the additional markers in this region we could decrease the smallest region of overlap for LOH between an intragenic marker in β-catenin (F Nollet et al, personal communication) and D3S1289. The distance between these markers is approximately

 © Cancer Research Campaign 1998

**Figure 2** Schematic representation of the regions on chromosomes 3, 11 and 17 with LOH. Patient identification numbers are above each row of symbols. ○, marker showing retention; ●, marker showing LOH; –, marker homozygous (not informative). Open spaces, not investigated. mi, microsatellite instability

15 cM. Part of the β-catenin gene is still in this region of smallest overlap.

### Chromosome 11q

The 34 tumours with LOH for chromosome 11q were tested for additional markers. Figure 3 shows the results of this screening. Among the markers tested, LOH was most frequent in these 34 tumours at D11S35 (79%), D11S938 (71%) and D11S528 (74%). The LOH pattern revealed three different regions with a high percentage deletion encompassing D11S35 on 11q22.1, D11S938 on 11q22.3 and D11S528 on 11q23.3 (see Figure 3).

### Chromosome 17p

On chromosome 17p13.3 a marker (D17S513) distal to the p53 tumour-suppressor gene had 38% LOH. TP53, a marker within the p53 coding region, had 20% LOH. In ten cases there is a

© Cancer Research Campaign 1998 

**Figure 3** Frequency of LOH observed for ten microsatellite markers for chromosome 11 in 34 cervical cancers

**Table 2** Comparison of β-catenin and E-cadherin expression compared with the LOH results on chromosome 3 and chromosome 16 respectively

| | LOH present, chromosome 3 | LOH absent, chromosome 3 |
|---|---|---|
| β-Catenin strong staining | 19 | 13 |
| β-Catenin weak staining | 5 | 1 |
| β-Catenin loss of staining | 1 | 0 |
| | LOH present chromosome 16 | LOH absent chromosome 16 |
| E-cadherin strong staining | 2 | 29 |
| E-cadherin weak staining | 3 | 3 |
| E-cadherin loss of staining | 0 | 0 |

breakpoint between D17S513 and TP53. One other marker in this area, D17S1537, was tested and had 32% LOH. This marker has not been mapped precisely, but based on our results it is most likely located distal to D17S513.

### Microsatellite instability

In addition to LOH, microsatellite instability was observed in 9 out of 64 cases ranging from 1–15 affected loci in 24 microsatellite markers, resulting in increased or decreased size of one or both alleles. All cases also had LOH at other loci. Two had microsatellite instability at three loci, whereas six cases were affected at only one locus. The remaining tumour (CT73) had microsatellite instability for almost all loci, except for one locus, D16S752, which had LOH (Figure 4).

### β-Catenin and E-cadherin expression

The β-catenin gene is located on chromosome 3p21 and an intragenic polymorphic β-catenin microsatellite marker shows frequent LOH. Therefore, β-catenin is a potential tumour-suppressor gene in cervical carcinomas. Inactivation of the β-catenin gene is expected to result in loss of expression of the β-catenin protein, and therefore we investigated β-catenin expression using immunohistochemistry. β-Catenin expression was strong in 32, weak in six cases and completely negative in one case (Table 2 and Figure 5). An inactivation of both β-catenin alleles would be expected to result in complete loss of β-catenin expression. We conclude that

**Figure 4** Microsatellite instability in patient 73. N, normal DNA; T, tumour DNA. Microsatellite markers used are shown above each pair of lanes. Arrows indicate the additional bands. For marker D16S752 this patient had LOH (arrow)

β-catenin inactivation is not frequent in cervical carcinomas and that for most cases in which chromosome 3p21.1 is lost, another tumour-suppressor gene may be inactivated. The one case with complete loss of β-catenin expression did show LOH on 3p21.1, and in this tumour the other β-catenin allele may be mutated.

β-Catenin associates with E-cadherin, which is located on chromosome 16q22.1. Therefore E-cadherin can be a candidate tumour-suppressor gene for the tumours showing LOH on chromosome 16, as has been established in other tumour types (Berx et al, 1995). E-cadherin expression was also studied in this tumour series. Strong E-cadherin expression was found in 31 of 37 cases, and six cases had weak expression. Complete absence of E-cadherin expression was never observed. There was no association between the level of β-catenin and E-cadherin expression.

### Correlation with tumour characteristics

In Table 3 the different clinical and histological parameters are correlated to losses on chromosome 3p, 11q and 17p. As can be seen in Table 3, the only significant correlation was for histological type and loss on chromosome 3p. LOH on chromosome 3p was observed in 54% of squamous carcinomas and in none of six adenocarcinomas. The LOH data were also correlated with respect to each other, but there is no correlation between the different losses.

### DISCUSSION

We have identified three chromosome regions that are likely to harbour tumour-suppressor genes important in the tumorigenesis of cervical cancer. Frequent LOH (41%) on chromosome 3p was found in the region between the β-catenin gene and marker D3S1289. Thus, we narrowed down the region of LOH to approximately 15 cM. The β-catenin gene is located in this smallest region of overlap. With the intragenic marker for β-catenin we found 37% LOH, which points to a possible role for this gene in cervical cancer. If the β-catenin gene would be inactivated by LOH and mutational inactivation, complete loss of β-catenin protein expression is expected. This was only found in one case. Mutation analysis by single-strand conformation polymorphism will be performed on the one tumour that had loss of β-catenin expression

© Cancer Research Campaign 1998

**Figure 5** Immunohistochemical staining for β-catenin and E-cadherin. Two different tumours are shown. (**A**) Strong membrane staining for β-catenin. (**B**) Tumour with loss of staining for β-catenin. (**C**) Strong membrane staining for E-cadherin. (**D**) Tumour with weak staining for E-cadherin. For both E-cadherin and β-catenin, staining is located at the plasma cell membrane. N, normal squamous epithelium; T, tumour tissue. Normal epithelium serves as an internal positive control for both β-catenin and E-cadherin staining and in the six cases with weak expression. In colon cancer and melanoma, it has been described that β-catenin can be stabilized and become unbound to APC by mutations in the tumour-suppressor gene APC or in β-catenin itself. If the association between APC and β-catenin is released, this will promote formation of β-catenin–Tcf complexes, which are translocated to the nucleus and activate gene transcription.

Various groups have studied chromosome 3p in cervical carcinomas, and 3p21–22 was a common region of LOH (Mullokandov et al, 1996). The marker most used and lost in this region is D3S2. It is located between D3S1289 and D3S1768, which we used. This region is also frequently involved in LOH in other tumours (breast, lung, kidney). In addition to the β-catenin gene, other candidate (tumour-suppressor) genes are located in this area, for instance TGM-4 (Gentile et al, 1995), which is the human prostate transglutaminase type IV gene whose function has been associated with the mammalian reproductive system and hMLH-1 (Bronner et al, 1994; Hemminki et al, 1994; Papadopolous et al, 1994), a human DNA mismatch repair gene involved in human non-polyposis colorectal cancer (HNPCC) families. However, LOH on 3p21–22 is not associated with the RER phenotype. Our results indicate that an as yet unidentified gene between β-catenin and D3S1289 most probably functions as a tumour-suppressor gene in cervical cancer that may also be involved in other tumour types. Further mapping will be required to identify this gene.

The E-cadherin gene is located on chromosome 16q22.1 and is a prime mediator of cell–cell adhesion in epithelial cells. This tumour-suppressor gene was studied because of its interaction with the β-catenin protein. We found six cases that had weak expression of E-cadherin; total loss of expression was not seen. LOH for 16q22.1 was sporadically found (10–14%) in the 64 cases studied. Others (Inoue et al, 1992; Vessey et al, 1995) have studied the expression of E-cadherin in normal cervical epithelium, CIN lesions, cervical carcinomas and metastases. These groups found that there was altered expression of E-cadherin in some CIN lesions and some tumours. The results from the different groups and our results suggest that alterations in the E-cadherin gene are not of major importance in cervical carcinomas.

© Cancer Research Campaign 1998

**Table 3** Losses on chromosome 3p, 11q and 17p correlated with tumour characteristics

| | Chromosome 3p | | | Chromosome 11q | | | Chromosome 17p | | |
|---|---|---|---|---|---|---|---|---|---|
| | *n* | Per cent loss | *P*-value | *n* | Per cent loss | *P*-value | *n* | Per cent loss | *P*-value |
| FIGO-stage | | | | | | | | | |
| I | 51 | 51 | 0.62 | 49 | 51 | 0.51 | 48 | 42 | 1.00 |
| II | 13 | 38 | | 12 | 67 | | 11 | 45 | |
| Lymphnode | | | | | | | | | |
| + | 15 | 60 | 0.50 | 14 | 57 | 0.98 | 14 | 21 | 0.11 |
| – | 48 | 46 | | 46 | 52 | | 44 | 50 | |
| Tumour size | | | | | | | | | |
| < 3 cm | 31 | 52 | 0.9 | 31 | 45 | 0.29 | 30 | 43 | 0.96 |
| > 3 cm | 32 | 47 | | 29 | 62 | | 28 | 39 | |
| Vasoinvasion | | | | | | | | | |
| No | 39 | 56 | 0.23 | 38 | 58 | 0.73 | 36 | 27 | 0.29 |
| Little | 32 | 13 | | 11 | 45 | | 11 | 45 | |
| High | 10 | 40 | | 10 | 50 | | 10 | 20 | |
| Histology | | | | | | | | | |
| Squamous | 57 | 54 | 0.03 | 54 | 56 | 0.54 | 53 | 42 | 1.00 |
| Adeno | 6 | 0 | | 6 | 33 | | 5 | 40 | |

We made a more specified LOH map for cervical cancer on chromosome 11q between 11q13.1 and 11q23.3 using twelve microsatellite markers. We have found three different markers with frequent LOH, D11S35, D11S938 and D11S528, but we could not find a single smallest region of overlap. These three markers are located on different chromosome bands. It is possible that three different loci on this chromosome are involved in cervical cancer. Hampton et al (1994) described about 44% loss of the region 11q22–q24 but used only five markers in this area, which are located in the same region as the markers used by us. Bethwaite et al (1995) used only one marker (D11S29) in this area, which had 30% loss. This marker is located at the same chromosome region as D11S528 used in these experiments. The region between 11q22–q24 is also involved in other tumours, including malignant melanoma (Tomlinson et al, 1993), breast cancer, colorectal cancer (Keldysh et al, 1993), ovarian cancer (Foulkes et al, 1993) and paragangliomas (Devilee et al, 1994). The STMY gene is located near marker D11S35 and is a candidate tumour-suppressor gene. A candidate locus near D11S938 is the PGL-locus. Candidate tumour-suppressor genes near D11S528 are NCAM and CD3D.

On chromosome 17, we studied the regions containing the p53 gene and BRCA-1. 17q23, containing BRCA-1, had LOH in only 4% of tumours and is not likely to be of importance in cervical carcinomas. TP53, in the p53 gene, had LOH in 20% of tumours which is in the same range found by others (Mitra et al, 1994; Mullokandov et al, 1996). In contrast, frequent LOH was found on 17p13.3 with two markers D17S513 (38%) and D17S1537 (32%). Park et al (1995) found 40% LOH with one of two markers (D17S34 and D17S5) used on 17p13.3. These markers are placed approximately 19 cM from our markers. This region also shows frequent LOH in a number of different tumours, such as carcinomas of the bladder, ovary, breast, malignant medulloblastomas, hepatocellular and medulloblastomas (Cornelis et al, 1994; Morris et al, 1995). Morris et al (1995) identified the CRK and ABR genes in this region; these genes are involved in signal transduction. Furthermore, Makos Wales et al (1995) located a new candidate suppressor gene in this region, HIC-1 (hypermethylated in cancer 1). This gene contains a p53-binding site, and is activated by wild-type p53. It is expressed in normal tissues, but expressed at a lower level in tumour cells in which it is hypermethylated (Makos Wales et al, 1995). In one study of breast cancer, the independent loss of 17p13.3 alleles was accompanied by increased levels of p53 mRNA, which suggests that the 17p13.3 tumour-suppressor gene may regulate p53 expression (Coles et al, 1990). From our results and the results of others it is clear that a tumour-suppressor gene that is important for cervical carcinomas must be present on chromosome 17p13.3.

Recently, it was found that tumours can have a RER (replication error) phenotype, pointing to malfunctioning of the DNA repair and replication mechanisms. Clearly, RER-positive tumours are defined as tumours that have microsatellite instability in at least two out of the seven microsatellite loci (Burks et al, 1994). One tumour in our series meets these criteria. RER is often found in colorectal cancers, colon, endometrial (Burks et al, 1994) and ovarian cancers (Orth et al, 1994; Liu et al, 1995). In cervical cancer, two groups (Mitra et al, 1995; Larson et al, 1996) reported microsatellite instability. Mitra et al (1995), reported this phenomenon only at a few loci on chromosome 5. Larson et al (1996) studied three different chromosomes in 89 primary cervical tumours and found RER+ phenotypes in 5.6% of cases. In our series, we found nine cases (14%) that had microsatellite instability at various loci, and LOH was shown in all these cases.

In conclusion, we have provided additional evidence for the probable presence of at least three tumour-suppressor genes in squamous cell carcinomas of the uterine cervix: one at 3p21, one at 17p13.3 and one to possibly three at chromosome 11. We will continue mapping the smallest region of LOH in these tumours and analyse candidate genes present in the regions of LOH. We have confirmed the presence of microsatellite instability in approximately 14% of cervical carcinomas.

*© Cancer Research Campaign 1998*

### ACKNOWLEDGEMENTS

We are grateful to Friedel Nollet University of Ghent, Belgium, for providing us with the intragenic β-catenin primers.

This study was supported by Stichting Vanderes.

### ABBREVIATIONS

LOH, loss of heterozygosity; RER, replication error; HPV, human papillomavirus; PCR, polymerase chain reaction; HNPCC, human non-polyposis colorectal cancer.

### REFERENCES

Bailey A, Norris AL, Leek JP, Clissold PM, Carr IM, Ogilvie DJ, Morrison JFJ, Meredith DM and Markham AF (1995) Yeast artificial chromosome cloning of the beta-catenin locus on human chromosome 3p21–22. *Chrom Res* **3**: 201–203

Berx G, Cleton-Jansen AM, Nollet F, De Leeuw WJF, Van De Vijver MJ, Cornelisse CJ and Van Roy F (1995) E-cadherin is a tumour/invasion suppressor gene mutated in human lobular breast cancers. *EMBO J* **14**: 6107–6115

Bethwaite PB, Koreth J, Herrington CS and McGee JO (1995) Loss of heterozygosity occurs at the D11S29 locus on chromosome 11q23 in invasive cervical carcinoma. *Br J Cancer* **71**: 814–818

Bronner CE, Baker SM, Morrison PT, Warren G, Smith LG, Lescoe MK, Kane M, Earabino C, Lipford J, Lindblom A, Tannergard P, Bollag RJ, Godwin AR, Ward DC, Nordenskjold M, Fishel R, Kolodner R and Liskay RM (1994) Mutation in the DNA mismatch repair gene homologue hMLH-1 is associated with hereditary non-polyposis colon cancer. *Nature* **368**: 258–261

Burks RT, Kessis TD, Cho KR and Hedrick L (1994) Microsatellite instability in endometrial carcinoma. *Oncogene* **9**: 1163–1166

Cattoretti G, Pileri S, Parravicini C, Becker MHG, Poggi S, Bifulco C, Key G, D'Amato L, Sabattini E, Feudale E, Reynolds F, Gerdes J and Rilke F (1993) Antigen unmasking on formalin-fixed, paraffin-embedded tissue sections. *J Pathol* **171**: 83–98

Chung GTY, Huang DP, Wai Lo K, Chan MKM, Wong FWS (1992) Genetic alterations in the carcinogenesis of cervical cancer. *Anticancer Res* **12**: 1485–1490

Coles C, Thompson AM, Elder PA, Cohen BB, Mackenzie IM, Cranston G, Chetty U, Mackay J, Macdonald M, Nakamura Y, Hoyheim B and Steel CM (1990) Evidence implicating at least two genes on chromosome 17p in breast carcinogenesis. *Lancet* **336**: 761–763

Cornelis RS, Vliet VM, Vos CBJ, Cleton-Jansen AM, Vijver VDJ, Peterse JL, Meera Khan P, Borresen AL, Cornelisse CJ and Devilee P (1994) Evidence for a gene on 17p13.3, distal to p53, as a target for allele loss in breast tumors without p53 mutations. *Cancer Res* **54**: 4200–4206

Devilee P, Schothorst EM, Bardoel AFJ, Bonsing BA, Kuipers-Dijkshoorn NJ, James MR, Fleuren GJ, Van Der Mey AGL and Cornelisse CJ (1994) Allelotype of head and neck paragangliomas: allelic imbalance is confined to the long arm of chromosome 11, the site of the predisposing locus PGL. *Genes Chrom Cancer* **11**: 71–78

Foulkes WD, Campbell IG, Stamp GWH and Trowsdale J (1993) Loss of heterozygosity and amplification on chromosome 11q in human ovarian cancer. *Br J Cancer* **67**: 268–273

Fujita M, Inoue M, Tanizawa O, Iwamoto S and Enomoto T (1992) Alterations of the p53 gene in human primary cervical carcinoma with and without human papillomavirus infection. *Cancer Res* **52**: 5323–5328

Gentile V, Grant FJ, Porta R and Baldini A (1995) Localization of the human prostate transglutaminase (type IV) gene (TGM4) to chromosome 3p21.33–p22 by fluorescence in situ hybridization. *Genomics* **27**: 219–220

Hampton GM, Penny LA, Baergen RN, Larson A, Brewer C, Liao S, Busby-Earle RMC, Williams AWR, Steel CM, Bird CC, Stanbridge EJ and Evans GA (1994) Loss of heterozygosity in cervical carcinoma: Subchromosomal localization of a putative tumor-suppressor gene to chromosome 11q22–q24. *Proc Natl Acad Sci USA* **91**: 6953–6957

Havre PA, Yuan J, Hedrick L, Cho KR and Glazer PM (1995) p53 inactivation by HPV16 E6 results in increased mutagenesis in human cells. *Cancer Res* **55**: 4420–4424

Hemminki A, Pel Tomaki P, Mecklin JP, Jarvinen H, Salovaara R, Nystrom-Lahti M, De La Chapelle A and Aaltonen LA (1994) Loss of the wild type MLH1 gene is a feature of hereditary nonpolyposis colorectal cancer. *Nature Genet* **8**: 405–410

Hengel JV, Nollet F, Berx G, Roy NV, Speleman F and Roy FV (1995) Assignment of the human beta-catenin gene (CTNNB1) to 3p22–p21.3 by fluorescence in situ hybridization. *Cytogenet Cell Genet* **70**: 68–70

Hoppe-Seyler F and Butz K (1995) Molecular mechanisms of virus-induced carcinogenesis: the interaction of viral factors with cellular tumor suppressor proteins. *J Mol Med* **73**: 529–538

Hulsken J, Birchmeier W and Behrens J (1994) E-cadherin and APC compete for the interaction with beta-catenin and the cytoskeleton. *J Cell Biol* **127**: 2061–2069

Inoue M, Ogawa H, Miyata M, Shiozaki H and Tanizawa O (1992) Expression of E-cadherin in normal, benign, and malignant tissues of female genital organs. *Am J Clin Pathol* **98**: 76–80

Isola J, Devries S, Chu L, Ghazvini S and Waldman F (1994) Analysis of changes in DNA sequence copy number by comparative genomic hybridization in archival paraffin-embedded tumor samples. *Am J Pathol* **145**: 1301–1308

Jiko K, Tsuda H, Sato S and Hirohashi S (1994) Pathogenetic significance of P53 and c-Ki-ras gene mutations and human papillomavirus DNA integration in adenocarcinoma of the uterine cervix and uterine isthmus. *Int J Cancer* **59**: 601–606

Jones MH and Nakamura Y (1992) Deletion mapping of chromosome 3p in female genital tract malignancies using microsatellite polymorphisms. *Oncogene* **7**: 1631–1634

Jou SS, Stewart DB, Stappert J, Nelson WJ and Marrs JA (1995) Genetic and biochemical dissection of protein linkages in the cadherin–catenin complex. *Proc Natl Acad Sci USA* **92**: 5067–5071

Kaelbling M, Burk RD, Atkin NB, Johnson AB and Klinger HP (1992) Loss of heterozygosity on chromosome 17p and mutant p53 in HPV-negative cervical carcinomas. *Lancet* **340**: 140–142

Karlsen F, Rabbitts PH, Sundresan V and Hagmar B (1994) PCR-RFLP studies on chromosome 3p in formaldehyde-fixed paraffin-embedded cervical cancer tissues. *Int J Cancer* **58**: 787–792

Kawanishi J, Kato J, Sasaki K, Fujii S, Watanabe N and Niitsu Y (1995) Loss of E-cadherin dependent cell–cell adhesion due to mutation of the beta-catenin gene in a human cancer cell line, HSC-39. *Mol Cell Biol* **15**: 1175–1181

Keldysh PL, Dragani TA, Fleischman EW, Konstantinova LN, Perevoschikov AG, Pierotti MA, Della Porta G and Kopnin BP (1993) 11q deletions in human colorectal carcinomas: Cytogenetics and restriction fragment length polymorphism analysis. *Genes Chrom Cancer* **6**: 45–50

Kohno T, Takayama H, Hamaguchi M, Takano H, Yamaguchi N, Tsuda H, Hirohashi S, Vissing H, Shimizu M, Oshimura M and Yokota J (1993) Deletion mapping of chromosome 3p in human uterine cervical cancer. *Oncogene* **8**: 1825–1832

Korinek V, Barker N, Morin JM, Van Wichen D, De Weger R, Kinzler KW, Vogelstein B and Clevers H (1997) Constitutive transcriptional activation by a β-catenin-Tcf complex in $APC^{-/-}$ colon carcinoma. *Science* **275**: 1784–1787

Kraus C, Liehr T, Hulsken J, Behrens J, Birchmeier W, Grzeschik KH and Ballhausen WG (1994) Localization of the human beta-catenin gene (CTNNB1) to 3p21: A region implicated in tumor development. *Genomics* **23**: 272–274

Larson AA, Kern S, Sommers RL, Yokota J, Cavenee WK and Hampton GM (1996) Analysis of replication error (RER+) phenotypes in cervical carcinoma. *Cancer Res* **56**: 1426–1431

Liu B, Nicolaides NC, Markowitz S, Willson JKV, Parsons RE, Jen J, Papadopolous N, Peltomaki P, De La Chapelle A, Hamilton SR, Kinzler KW and Vogelstein B (1995) Mismatch repair gene defects in sporadic colorectal cancers with microsatellite instability. *Nature Genet* **9**: 48–55

Makos Wales M, Biel MA, El Deiry W, Nelkin BD, Issa JP, Cavenee WK, Kuerbitz SJ and Baylin SB (1995) p53 activates expression of HIC-1, a new candidate tumour suppressor gene on 17p13.3. *Nature Med* **1**: 570–577

Mansur CP, Marcus B, Dalal S and Androphy EJ (1995) The domain of p53 required for binding HPV 16 E6 is separable from the degradation domain. *Oncogene* **10**: 457–465

Misra BC and Srivatsan ES (1989) Localization of HeLa cell tumor-suppressor gene to the long arm of chromosome 11. *Am J Hum Genet* **45**: 565–577

Mitra AB, Murty VVVS, Li RG, Pratap M, Luthra UK and Chaganti RSK (1994) Allelotype analysis of cervical carcinoma. *Cancer Res* **54**: 4481–4487

Mitra AB, Murty VVVS, Singh V, Li RG, Pratap M, Sodhani P, Luthra UK and Chaganti RSK (1995) Genetic alterations at 5p15: A potential marker for progression of precancerous lesions of the uterine cervix. *J Natl Cancer Inst* **87**: 742–745

Morin PJ, Sparks AB, Korinek V, Barker N, Clevers H, Vogelstein B and Kinzler KW (1997) Activation of β-catenin-Tcf signalling in colon cancer by mutations in β-catenin or APC. *Science* **275**: 1787–1790

© Cancer Research Campaign 1998 

Morris C, Benjes S, Haataja L, Ledbetter DH, Heisterkamp N and Groffen J (1995) Spatial organization of ABR and CRK genes on human chromosome band 17p13.3. *Oncogene* **10**: 1009–1011

Mullokandov MR, Kholodilov NG, Atkin NB, Burk RD, Johnson AB and Klinger HP (1996) Genomic alterations in cervical carcinoma: Losses of chromosome heterozygosity and human papilloma virus tumor status. *Cancer Res* **56**: 197–205

Nollet F, Berx G, Molemans F and Van Roy F (1996) Genomic organization of the human beta-catenin gene (CTNNBI). *Genomics* **32**: 413–424

Orth K, Hung J, Gazdar A, Bowcock A, Mathis JM and Sambrook J (1994) Genetic instability in human ovarian cancer cell lines. *Proc Natl Acad Sci USA* **91**: 9495–9499

Papadopolous N, Nicolaides NC, Wei YF, Ruben SM, Carter KC, Rosen CA, Haseltine WA, Fleischmann RD, Fraser CM, Adams MD, Venter JC, Hamilton SR, Petersen GM, Watson P, Lynch HT, Peltomaki P, Mecklin JP, De La Chapelle A, Kinzler KW and Vogelstein B (1994) Mutation of a mutL homolog in hereditary colon cancer. *Science* **263**: 1625–1629

Park SY, Kang YS, Kim BG, Lee SH, Lee ED, Lee KH, Park KB and Lee JH (1995) Loss of heterozygosity on the short arm of chromosome 17 in uterine cervical carcinomas. *Cancer Genet Cytogenet* **79**: 74–78

Rader JS, Kamarasova T, Huettner PC, Li L, Li Y and Gerhard DS (1996) Allelotyping of all chromosomal arms in invasive cervical cancer. *Oncogene* **13**: 2737–2741

Rubinfeld B, Souza B, Albert I, Muller O, Chamberlain SH, Masiarz FR, Munemitsu S and Polakis P (1993) Association of the APC gene product with beta-catenin. *Science* **262**: 1731–1734

Rubinfeld B, Souza B, Albert I, Munemitsu S and Polakis P (1995) The APC protein and E-cadherin form similar but independent complexes with alfa-catenin, beta-catenin and plakoglobin. *J Biol Chem* **270**: 5549–5555

Rubinfeld B, Robbins P, El-Gamil M, Albert I, Porfiri E and Polakis P (1997) Stabilization of β-catenin by genetic defects in melanoma cell lines. *Science* **257**: 1790–1792

Saxon PJ, Srivatsan ES and Stanbridge EJ (1986) Introduction of human chromosome 11 via microcell transfer controls tumorigenic expresson of HeLa cells. *EMBO J* **5**: 3461–3466

Schneider J, Rubio MP, Rodriguez-Escudero FJ, Seizinger BR and Castresana JS (1994) Identification of p53 mutations by means of single strand conformation polymorphism analysis in gynaecological tumours: Comparison with the results of immunohistochemistry. *Eur J Cancer* **30A**: 504–508

Su LK, Vogelstein B and Kinzler KW (1993) Association of the APC tumor suppressor protein with catenins. *Science* **262**: 1734–1737

Tomlinson IPM, Gammack AJ, Stickland JE, Mann GJ, Mackie RM, Kefford RF and McGee JO (1993) Loss of heterozygosity in malignant melanoma at loci on chromosomes 11 and 17 implicated in the pathogenesis of other cancers. *Genes Chrom Cancer* **7**: 169–172

Trent JM, Wiltshire R, Su LK, Nicolaides NC, Vogelstein B and Kinzler KW (1995) The gene for the APC-binding protein beta-catenin (CTNNB1) maps to chromosome 3p22, a region frequently altered in human malignancies. *Cytogenet Cell Genet* **71**: 343–344

Vessey CJ, Wilding J, Folarin N, Hirano S, Takeichi M, Soutter P, Stamp GWH and Pignatelli M (1995) Altered expression and function of E-cadherin in cervical intraepithelial neoplasia and invasive squamous cell carcinoma. *J Pathol* **176**: 151–159

Yokota J, Tsukada Y, Nakajima T, Gotoh M, Shimosato Y, Mori N, Tsunokawa Y, Sugimura T and Terada M (1989) Loss of heterozygosity on the short arm of chromosome 3 in carcinoma of the uterine cervix. *Cancer Res* **49**: 3598–3601

© Cancer Research Campaign 1998